# Inefficacy of neck cooling in suppressing core body temperature elevation during exercise in a hot environment: a randomized cross-over trial

**DOI:** 10.1265/ehpm.25-00041

**Published:** 2025-07-30

**Authors:** Kotaro Ishizuka, Chikage Nagano, Mai Togawa, Kentaro Kado, Keiichi Tajima, Kimiyo Mori, Seichi Horie

**Affiliations:** 1 Department of Health Policy and Management, Institute of Industrial Ecological Sciences, University of Occupational and Environmental Health (UOEH), Japan, 1-1 Iseigaoka, Yahatanishi-ku, Kitakyushu-City, Fukuoka, Japan; 2 Asahi Kasei Corporation, 1-2 Yurakucho 1-chome, Chiyoda-ku, Tokyo, Japan; 3 USJ LLC, 2-1-33 Sakurajima, Konohana-ku, Osaka-City, Osaka, Japan

**Keywords:** Heat-related illnesses, Neck cooling, Partial body cooling, Core temperature, Per-cooling

## Abstract

**Background:**

Neck cooling is a practical method for preventing heat-related illness, however, its effectiveness in general workers is not well established. This study aimed to assess the effects of neck cooling on core body temperature and other physiological markers during exercise in a hot environment.

**Methods:**

This randomized crossover trial was conducted from November 2023 to April 2024 at the Shared-Use Research Center at UOEH. Fourteen healthy adult males participated in the study under two conditions: with neck cooling (COOL) and without neck cooling (CON). All participants completed both conditions, and the order of condition assignment was determined by a random draw. Participants first rested for 10 minutes in a 28.0 °C, 50% relative humidity environment, followed by a rest in a 35.0 °C, 50% relative humidity environment for another 10 minutes. In the COOL condition, participants wore a neck cooler containing 1,200 g of ice while exercising at 50% Heart Rate Reserve on a bicycle ergometer for 20 minutes. Afterward, they rested for 15 minutes in the hot environment while still wearing the cooler.

**Main outcome measures:**

Core body temperature (rectal and esophageal), forehead skin temperature, and heart rate were continuously monitored and compared using a mixed model. Estimated sweat volume was calculated based on changes in body weight before and after the experiment.

**Results:**

At the end of the rest period, no significant differences were observed between the COOL and CON conditions in rectal temperature (37.76 ± 0.18 °C versus 37.75 ± 0.24 °C, p = 0.9493), esophageal temperature (37.75 ± 0.30 °C versus 37.76 ± 0.23 °C, p = 0.7325), forehead skin temperature (36.87 ± 0.29 °C versus 36.88 ± 0.27 °C, p = 0.2160), or heart rate (104.18 ± 7.56 bpm versus 107.52 ± 7.40 bpm, p = 0.1035). Estimated sweat loss was similar between conditions (578 ± 175 g for CON versus 572 ± 242 g for COOL, p = 0.5066). While more participants felt cooler in the COOL condition, RPE showed no significant difference.

**Conclusion:**

Neck cooling did not significantly affect core temperature or perceived exertion. Maintaining close contact with the skin at sufficiently low temperatures or utilizing cooling methods that prevent excessive negative feedback may be necessary to enhance the effectiveness of neck cooling.

## Graphical Abstract

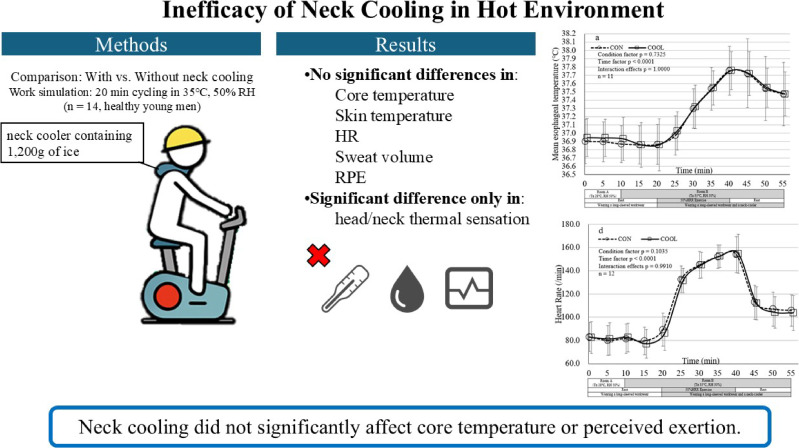

## Background

In Japan, heat-related illnesses frequently occur, particularly during the summer season. In 2023, the number of occupational deaths and cases requiring four or more days of leave due to heat-related illnesses totaled 1,106 [1], indicating that heat-related illnesses during physical labor in hot environments have become a critical issue in the workplace. The Ministry of Health, Labour and Welfare has emphasized in its 14th Occupational Accident Prevention Plan [2] the necessity to prevent the occurrence of heat-related illnesses in the workplace and to confirm and disseminate response measures when such illnesses occur. Therefore, it is crucial to implement various preventive measures in the labor environment.

To prevent heat-related illnesses, measures must be taken at three stages: pre-cooling (before work), per-cooling (during work), and post-cooling (after work). Research results have been reported for both pre-cooling [3, 4] and post-cooling [5–7] interventions for the general population, including workers. Regarding per-cooling, Bongers et al. [8] conducted a review of several studies involving athletes and demonstrated the utility of per-cooling by showing improved exercise performance. However, their findings revealed that core body temperature did not always decrease. A review by Cao et al. [9] on head and neck cooling during athletes’ exercise reported varying results on core body temperature changes, indicating that the effects of per-cooling on core body temperature are not fully understood. Furthermore, Chicas et al. [10] noted that very little is known about cooling interventions in occupational settings, and they highlighted that it is challenging to apply the findings from studies involving athletes to the general worker population due to differences in physical activity, workload, and environmental conditions. They emphasized the need for further research to validate the effectiveness and practicality of cooling interventions for general workers.

Among practical per-cooling methods that can be implemented during work are electric fan-equipped workwear, phase change material (PCM) packs, cooling garments with refrigerant, and water-cooled cooling garments [11, 12]. These methods have been recommended by the Ministry of Health, Labour and Welfare’s “Cool Work Campaign” [13] and are gradually being adopted in Japanese workplaces. However, electric fan-equipped workwear is often unsuitable for environments where workers are exposed to dust or chemicals, as it could increase exposure risks. Regarding cooling garments, Inoue et al. [14] pointed out that increasing the cooling range to enhance the cooling effect results in increased weight due to the addition of water or refrigerants, which in turn increases the physical burden on workers. Additionally, while water-cooled garments offer superior cooling effects, they may restrict movement due to hoses, potentially impairing work efficiency. Their high cost also presents challenges for implementation in the workplace. Considering these factors, per-cooling methods that maintain work efficiency and are affordable and easy to introduce into labor environments are necessary. Therefore, it is important to explore whether localized cooling of limited body surface areas can effectively cool the body for general workers.

We focused on neck cooling as a potential solution for localized cooling. Several studies have reported on the effectiveness of cooling different body parts, such as the forearms [15, 16], palms [17, 18], and lower legs [19]. For instance, Yeargin et al. [20] reported that cooling from the humerus epicondyle to the distal metacarpal for 15 minutes with 5 °C water in eight firefighters improved gastrointestinal temperature (T_GI_) and the physiological strain index. House et al. [19] reported the effectiveness of cooling the palms, feet, forearms, and lower legs using 10 °C water for 60 minutes over personal protective equipment (PPE) in a supine position. However, many reports on localized cooling involve the use of large amounts of cold water or required equipment, making it impractical for many work environments. Implementing these body parts during work may not be realistic, as it is cumbersome for workers who need to wear gloves, long sleeves, and protective gear, which can reduce work efficiency. Moreover, cooling these body parts during work may not be realistic.

There is one study on neck cooling in an agricultural setting by Choi et al. [21], who reported that rectal temperature did not decrease with neck cooling alone. However, the workload in this study was lower than that of walking or running. Moreover, gel packs used for neck cooling melted within about an hour, meaning cooling was insufficient for the 2-hour experiment duration, raising doubts about whether the effectiveness of neck cooling was adequately evaluated. We have checked other reports of neck cooling but have not found any reports for general workers.

The neck area is known to be advantageous for cooling the body because the internal carotid artery and jugular vein run close to the surface, allowing for the efficient cooling of arterial and venous blood. Additionally, the head and neck area are more heat-sensitive compared to the trunk, making cervical cooling more effective in alleviating heat stress. Thus, the cervical region is recognized as a favorable site for body cooling [22]. In recent years, various products for direct neck cooling have been introduced to the Japanese market for heat illness prevention. These products are increasingly used not only by workers but also by the general public, leading to market expansion. Many workers use these products in their workplaces because they are easy to implement and manage. However, as mentioned earlier, there is limited evidence on the effectiveness of neck cooling for general workers. Therefore, this study adopted a neck cooler capable of covering the entire neck with ice as the cooling method. This approach was selected because, among commercially available neck cooling products, it is cost-effective, simple to prepare using just a freezer to make ice, and does not require electricity, making it suitable for a wide range of workplaces. The aim of this study was to evaluate the preventive effects of neck cooling on heat-related illnesses during exercise in hot environments, from the start of physical activity to the end of post-exercise rest.

## Methods

### Participants

Based on our laboratory’s previous research data, we found that participants’ core temperatures exhibited a standard deviation of about 0.3 °C. In this study, we aimed to detect a mean difference of at least 0.3 °C between conditions, with a two-sided significance level of 5% and a statistical power of 80%. The required sample size was calculated to be 10 participants. Anticipating a dropout rate of approximately 20%, we recruited fourteen healthy young men to represent workers in hot environments in Japan. All participants were asked to carefully read a document explaining the purpose and procedures of the experiment, and all gave their written informed consent prior to participation. A questionnaire was used to confirm that participants had no history of cerebrovascular or cardiovascular diseases, smoking habits, or alcohol consumption on the previous day. Participants were instructed to refrain from intense exercise and alcohol consumption from the day before each experimental measurement until the start of the experiment.

### Measurements

Core body temperature, skin temperature, and estimated sweat loss were measured as primary outcomes, while subjective evaluations and ratings of perceived exertion (RPE) were assessed as secondary outcomes. All primary and secondary outcomes were predefined prior to the start of the experiment. Before the experiment, each participant’s VO_2max_ was measured using a breath-by-breath method with an automatic respiratory gas analyzer (ARCO-2000, Arco System Inc., Chiba, Japan) while exercising on a bicycle ergometer. The corresponding workload equivalent to 50% Heart Rate Reserve (HRR) was calculated for each participant.

Core body temperatures were monitored using esophageal (T_es_) and rectal (T_re_) thermometers. T_es_ was measured by inserting a thermocouple through the mouth, and T_re_ was measured by inserting a thermocouple into the rectum, covered with a disposable rubber sheath (Nikkiso-therm, Tokyo, Japan). The forehead skin temperature (T_fh_) was measured by attaching a thermocouple with medical tape to the right forehead. The thermocouples met Class 1 standards under JIS C1602-2015, with conductor materials consisting of copper and constantan (Ninomiya Electric Wire Co., Ltd., Kanagawa, Japan). The wire diameters were specified as 0.1 mm for T_es_ 0.32 mm for T_re_, and 0.2 mm for T_fh_, processed by the Shared-Use Research Center at UOEH. The heart rate (HR) was monitored using a heart rate monitor (BSM-2401, Nihon Kohden, Tokyo, Japan). Exercise intensity was evaluated using the Borg Scale for Rating of Perceived Exertion (RPE) (7 = Extremely light, 9 = Very light, 11 = Light, 13 = Somewhat hard, 15 = Hard, 17 = Very hard, 19 = Extremely hard). Thermal sensation for the head, neck, and entire body was assessed using a categorical scale based on ISO 10551, ranging from +5 (Extremely hot) to −5 (Extremely cold). Measurements were recorded at 20 minutes after the start of the experiment, immediately before exercise (Pre-Ex), 10 minutes after starting exercise (Ex-10), 20 minutes after starting exercise (Ex-20), and at the end of the experiment (End).

Estimated sweat volume was calculated by providing participants with pre-weighed towels and measuring their body weight before and after the experiment without any clothing. (measuring device: IS-150IGG-H, Sartorius Japan K.K., Tokyo, Japan).

The experiment was conducted from November 2023 to April 2024 in the Artificial Climate Chamber at the Cooperative Research Center, University of Occupational and Environmental Health, Japan (TBR-8E20W0P2T, Espec, Osaka, Japan). All participants consumed a 400 kcal meal (Calorie Mate, Otsuka Pharmaceutical, Tokyo, Japan) and 500 ml of water (CRYSTAL GEYSER, Crystal Geyser Water Company, Calistoga, CA, USA) 1 hour before the experiment. Water intake was restricted during the experiment. Participants wore long-sleeved workwear (VES2360 and VES360, Midorianzen, Tokyo, Japan), a 100% cotton short-sleeved undershirt, and socks.

To minimize circadian variation and avoid carry-over effects, all experiments were conducted at 1:00 pm with at least a 3-day interval between sessions. As this study employed a randomized crossover trial design, the order of condition assignment was determined by a random draw.

### Cooling conditions

**Fig. 1:**
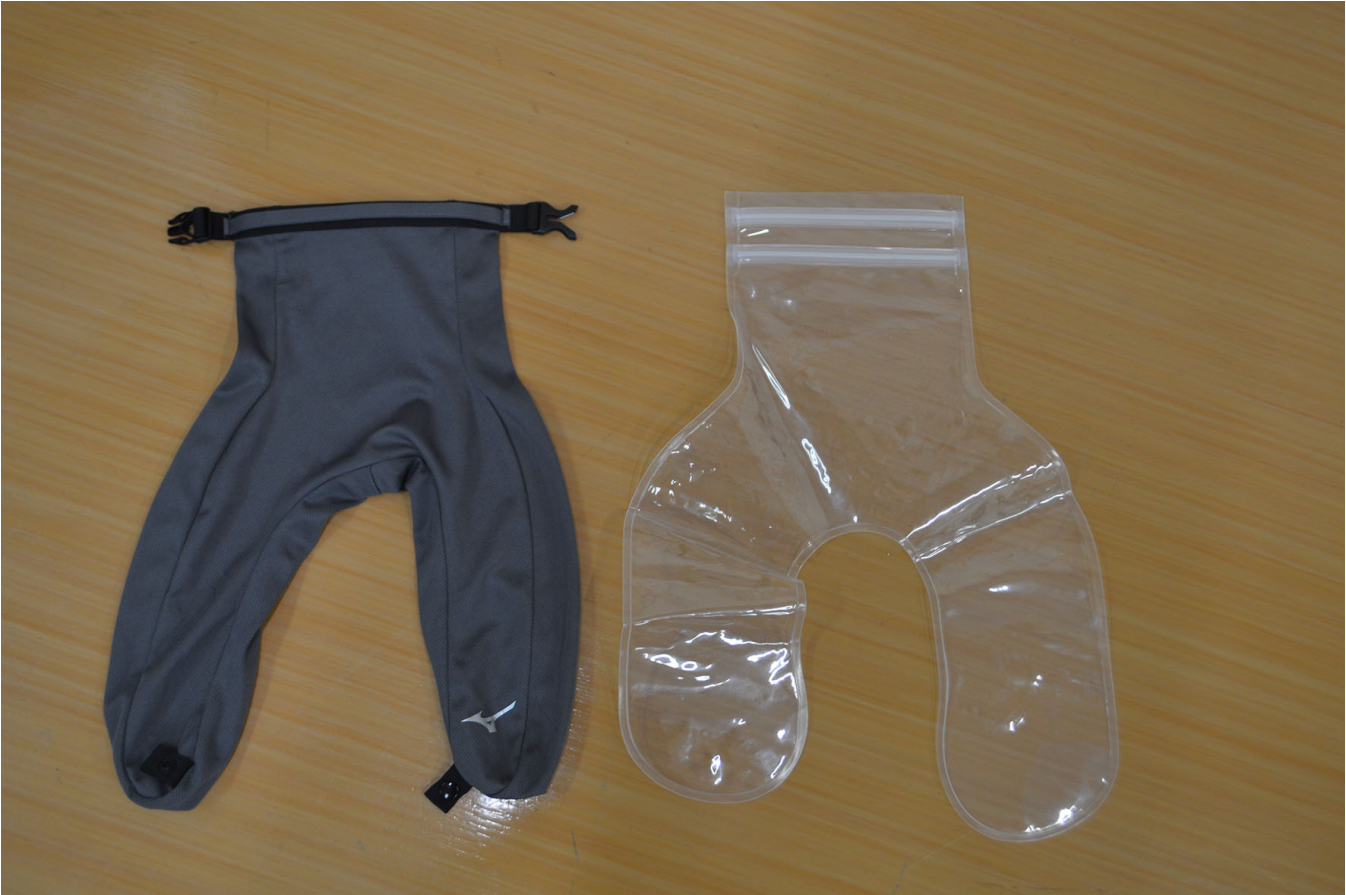
Neck Cooler. Left: Outer bag. Right: Inner bag.

In this experiment, a commercially available ice bag neck cooler (E2MYA01705, Mizuno Corporation, Osaka, Japan) (Fig. 1) designed for neck cooling was used. The neck cooler was placed over the shoulders from the back of the neck and secured at the front with a button, covering the lower half of the neck from the shoulder to the lower end of the thyroid cartilage, with a cooling surface area of 50 × 7 cm. The neck cooler was filled with ice to cool the participants. Ice cubes were made by filling a standard ice tray with tap water and freezing them in a household freezer (YFR-D111, Yamazen Corporation, Osaka, Japan). 60 ice cubes measuring approximately 3 × 3 × 2.5 cm each (a total of 1.2 kg) were prepared.

10 minutes after starting the experiment, the inner bag made of Ethylene-Vinyl Acetate Copolymer (EVA) resin was filled with only ice, set into the polyester outer bag, and kept in a cooler box until worn by participants to prevent melting ice. The temperature inside the neck cooler was monitored with a thermocouple during the experiment.

### Experimental procedures

**Fig. 2:**
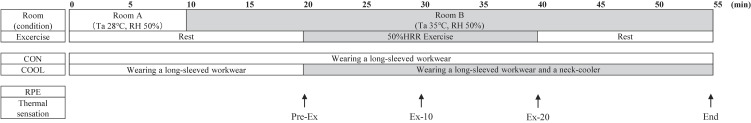
Schematic of the experimental protocol. In each group, subjects were interviewed using a subjective scale, at the following four time-points: immediately before exercise initiation (Pre-Ex); 10 min after exercise initiation (Ex-10); 20 min after exercise initiation (Ex-20); and test completion (End). CON, control condition; COOL, with neck cooling condition; HRR, Heart Rate Reserve; RH, Relative Humidity; RPE, Rating of perceived exertion; Ta, ambient temperature.

Figure 2 shows a schematic of the experimental protocol. After measuring their body weight, participants were fitted with all measurement equipment. They first rested for 10 minutes in a room with a dry bulb temperature of 28.0 °C and 50% relative humidity (Room A). They then moved to a room with a dry bulb temperature of 35.0 °C and 50% relative humidity (Room B) and rested for another 10 minutes. Just before the start of exercise, participants moved from the chair to the ergometer. In the COOL condition, the neck cooler was applied while participants were on the ergometer. In contrast, in the CON condition, no neck cooler was applied, and participants waited on the ergometer until the start of exercise.

20 minutes after the start of the experiment, participants began exercising on a bicycle ergometer at a constant pace for 20 minutes. The ergometer load was set to correspond to each participant’s 50% HRR. After the exercise, they got off the ergometer, and in the COOL condition, the neck cooler remained on while they rested for 10 minutes in Room B. The experiment ended after removing all monitoring devices and measuring body weight again. The experiment was halted if participants developed symptoms of heat illness or if rectal temperature reached 38.5 °C.

### Statistical analysis

Statistical analysis was performed using JMP Pro 18 (SAS Institute Inc., Cary, NC, USA). Data for core temperatures (T_re_, T_es_), skin temperature (T_fh_), and heart rate (HR) were recorded every 10 seconds, with analysis based on moving averages calculated over one-minute intervals. Average values for every minute of the 45-minute period from the start of exercise to the end of rest were analyzed. Standard deviations (SD) were calculated as one-minute averages for each minute. Differences in mean measurements between CON and COOL conditions, as well as across different time points, were analyzed using mixed models. These models included fixed effects for condition, time, and condition-by-time interaction, and a random effect for participants. For T_re_, T_es_, T_fh_, and HR, one-minute average values at five-minute intervals were used for analysis. For subjective evaluations, average values from each time point between Pre-Ex and Ex-20 were used. The estimated sweat volume for each participant was calculated from the change in body weight before and after the experiment using the following formula:

$$\begin{array}{rl} & \text{Estimated sweat volume}\\ & \;=\text{Body weight before the experiment}\\ & \;-\text{Body weight after the experiment} \end{array}$$

Furthermore, considering individual weight differences, the percentage change in body weight was calculated using the following formula:

$$\begin{array}{rl} & \text{\%Weight change}\\ & \;=(\text{Body weight before the experiment}\\ & \;-\text{Body weight after the experiment})\\ & \;/\text{Body weight before the experiment} \end{array}$$

Estimated sweat volume and %Weight change were analyzed using the Wilcoxon test. Statistical significance was set at p < 0.05.

## Results

Fourteen healthy adult males participated in the experiment (mean ± SD: age 23.2 ± 3.7 years; height 172.7 ± 6.8 cm; weight 66.0 ± 8.9 kg; VO_2max_ 36.8 ± 6.8 ml/kg/min). All participants completed both the CON and COOL conditions. Participant characteristics are shown in Table 1. One participant (E), whose difference in core temperature between the CON and COOL conditions at the start of the experiment was outside the mean ± 2SD range, was excluded from the analysis due to inconsistencies in experimental conditions. For esophageal temperature (T_es_), 2 participants (F, L) were excluded from the analysis because their esophageal temperature dropped by more than 0.5 °C during exercise, indicating the possibility that the thermocouple may have slipped out. Consequently, the statistical analysis for T_es_ was performed with data from 11 participants. One participant (C) was also excluded from the analysis of forehead skin temperature (T_fh_) because the thermocouple detached during the experiment, leaving 12 participants for the T_fh_ analysis. For Heart Rate, one participant (N) was excluded from the analysis because the difference between the CON and COOL conditions at the start of the experiment fell outside the mean ± 2SD range. Consequently, the statistical analysis for HR was performed with data from 12 participants. For estimated sweat volume, one participant (B) was excluded from the analysis because he drank water before the post-experiment body weight measurement, resulting in data from 12 participants being used for analysis.

**Table 1:** Participant characteristics

**Participant**	**Age** **(years)**	**Gender**	**Height** **(cm)**	**Weight** **(kg)**	**BMI**	**VO_2max_** **(ml/kg/min)**	**Order of the ** **condition**	**Interval between experiments (day)**
A	19	M	169	55.5	19.4	43	CON→COOL	8
B	19	M	179	63.6	19.8	43	CON→COOL	34
C	20	M	178	80.0	25.2	31	CON→COOL	33
D	21	M	168	55.5	19.7	37	CON→COOL	45
E	21	M	183	71.9	21.5	38	CON→COOL	60
F	21	M	173	75.5	25.2	22	CON→COOL	15
G	23	M	175	80.9	26.6	41	CON→COOL	3
H	26	M	165	72.0	26.4	40	CON→COOL	7
I	19	M	176	63.5	20.5	33	COOL→CON	26
J	23	M	183	67.4	20.1	31	COOL→CON	25
K	27	M	170	55.1	19.0	42	COOL→CON	8
L	29	M	176	65.4	21.1	48	COOL→CON	16
M	29	M	160	53.1	20.7	38	COOL→CON	41
N	30	M	163	65.0	24.5	28	COOL→CON	12

BMI, Body Mass Index; CON, Control condition; COOL, With neck cooling condition; M, Man; VO_2max_, Maximal oxygen consumption.

No frostbite was observed in the cooling areas of any participants under the COOL condition at the end of the experiment, and no participants reported any health issues due to cooling. The temperature inside the ice-filled neck cooler averaged 7.68 ± 5.13 °C throughout the experiment and remained constant until the end. At the end of the experiment, approximately half of the ice in the neck cooler had melted into liquid.

Table 2 presents the results of the measured indices for each condition.

**Table 2:** the results of the measured indices

**Measured indices**	**Condition**	**Timing of measurements**				**Condition factor** **p value**	**Time factor** **p value**	**Interaction effects** **p value**

		**The end of exercise**		**After experiment**				

		**Mean (95% CI)**	**Difference**	**Mean (95% CI)**	**Difference**			
Esophageal temperature (°C)^a^	CON	37.76 (37.60–37.91)	0.00	37.47 (37.27–37.67)	0.00	0.7325	<0.001	1.0000
	COOL	37.76 (37.76–37.75)		37.47 (37.48–37.47)				
Rectal temperature (°C)^a^	CON	37.55 (37.44–37.65)	0.06	37.76 (37.67–37.86)	0.02	0.9493	<0.001	0.7551
	COOL	37.49 (37.37–37.60)		37.75 (37.62–37.87)				
Forehead skin temperature (°C)^a^	CON	37.05 (36.94–37.16)	−0.04	36.87 (36.72–37.03)	−0.01	0.2160	<0.001	0.9944
	COOL	37.09 (36.94–37.24)		36.88 (36.74–37.02)				
Heart Rate (/min)^a^	CON	155.51 (147.03–163.99)	0.42	107.52 (100.12–114.92)	3.34	0.1035	<0.001	0.9910
	COOL	155.09 (146.50–163.68)		104.18 (96.63–111.74)				
Head and neck thermal sensation^a^	CON	4.23 (3.72–4.74)	3.46	2.69 (1.97–3.42)	2.62	<0.001	0.0025	0.3088
	COOL	0.77 (−0.18–1.71)		0.08 (−0.62–0.77)				
Body thermal sensation^a^	CON	4.38 (4.00–4.77)	0.77	2.92 (2.29–3.56)	0.62	0.0016	<0.001	0.8245
	COOL	3.62 (2.98–4.25)		2.31 (1.61–3.00)				
Rating of Perceived Exertion^a^	CON	14.85 (13.55–16.14)	1.00	10.92 (10.09–11.76)	0.31	0.4349	<0.001	0.0711
	COOL	13.85 (12.78–14.91)		10.62 (9.58–11.65)				
Estimated sweat volume (g)^b^	CON			578.25 (486.50–670.00)	6.00	0.5066		
	COOL			572.25 (445.39–699.11)				
% Weight change (%)^b^	CON			0.87 (0.76–0.98)	0.01	0.7950		
	COOL			0.86 (0.70–1.02)				

CI, confidence interval; CON, Control condition (without neck cooling condition); COOL, With neck cooling condition.

a: Analyzed using mixed models, with fixed effects for condition, time, and condition-by-time interaction, and a random effect for participants.

b: Analyzed using the Wilcoxon test.

### Core temperature, skin temperature, and heart rate

**Fig. 3:**
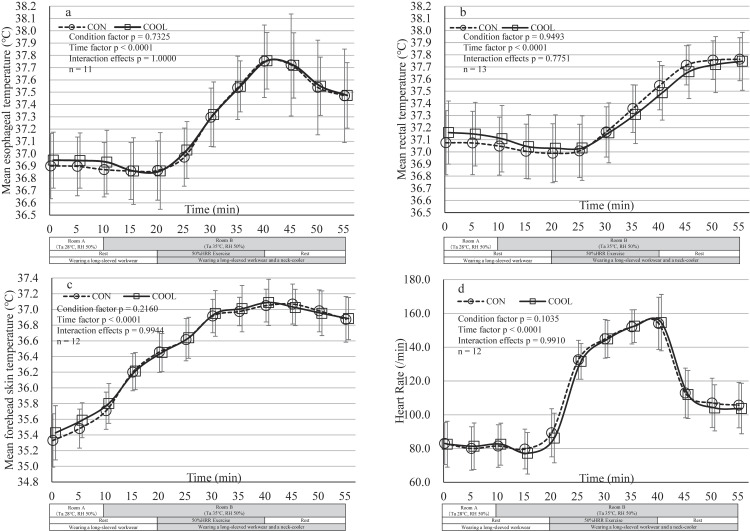
Trends of Physiological Parameters in CON and COOL Conditions. (a) Mean esophageal temperature. (b) Mean rectal temperature. (c) Mean forehead skin temperature. (d) Mean heart rate and changes. The Room A was maintained at 28 °C ambient temperature and 50% relative humidity, and the Room B at 35 °C ambient temperature and 50% relative humidity. Values are mean ± SD (n = 11 for esophageal temperature, n = 13 for rectal temperature, and n = 12 for forehead skin temperature and heart rate). CON, control condition; COOL, neck cooling condition; HRR, Heart Rate Reserve; RH, Relative Humidity; SD, standard deviation; Ta, ambient temperature. No significant differences were observed between CON and COOL conditions in a mixed model.

Esophageal temperature, rectal temperature, forehead skin temperature, and heart rate all increased during exercise and showed a downward trend during the post-exercise rest period, following expected physiological patterns. For all variables, the time factor was significant, indicating changes over time; however, no significant differences were observed between the CON and COOL conditions, and no significant interaction effects were found (Fig. 3).

### Estimated sweat volume

**Fig. 4:**
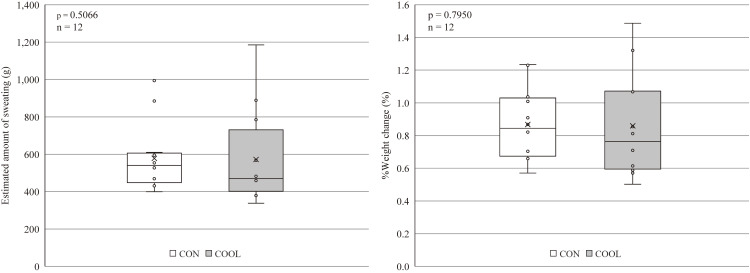
Estimated Sweat Volume. The estimated sweat volume for each participant is shown using a box plot. The left figure shows the changes in participants’ body weight before and after the experiment. The right figure shows %weight change, accounting for the differences in participants’ body weight. (n = 12). CON, control condition; COOL, with neck cooling condition. No significant differences in the Wilcoxon test.

Estimated sweat volume and %Weight change were similar between conditions, with no statistically significant effect of condition observed (Fig. 4).

### Subjective evaluations

**Fig. 5:**
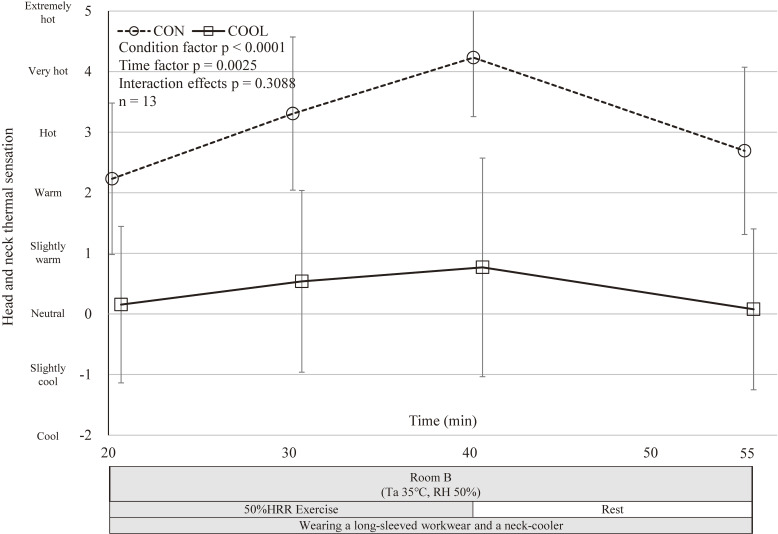
Trends of head and neck thermal sensation. Mean head and neck thermal sensation in CON and COOL conditions; 20 min, immediately before exercise initiation; 30 min, 10 min after exercise initiation; 40 min, 20 min after exercise initiation; 55 min, test completion. Values are mean ± SD (n = 13). CON, control condition; COOL, with neck cooling condition; RH, Relative Humidity; SD, standard deviation; Ta, ambient temperature. *Different (p < .05) in a mixed model.

**Fig. 6:**
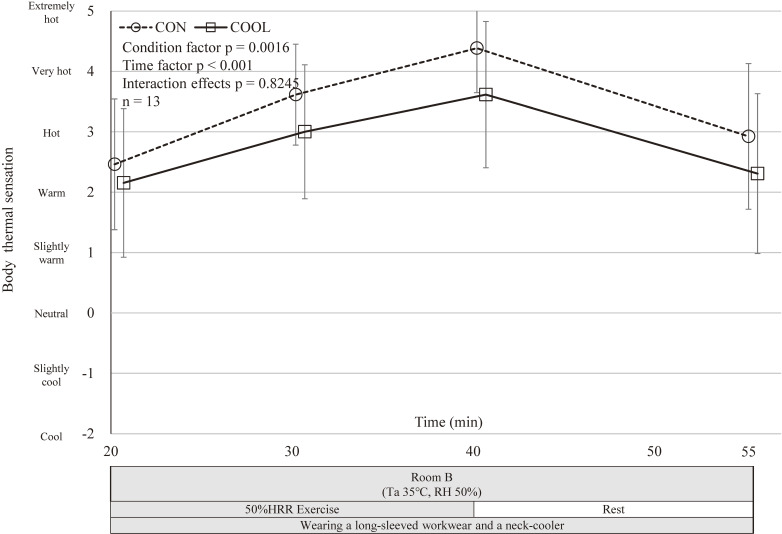
Trends of body thermal sensation. Mean body thermal sensation in CON and COOL conditions; 20 min, immediately before exercise initiation; 30 min, 10 min after exercise initiation; 40 min, 20 min after exercise initiation; 55 min, test completion. Values are mean ± SD (n = 13). CON, control condition; COOL, with neck cooling condition; RH, Relative Humidity; SD, standard deviation; Ta, ambient temperature. No significant differences in a mixed model.

**Fig. 7:**
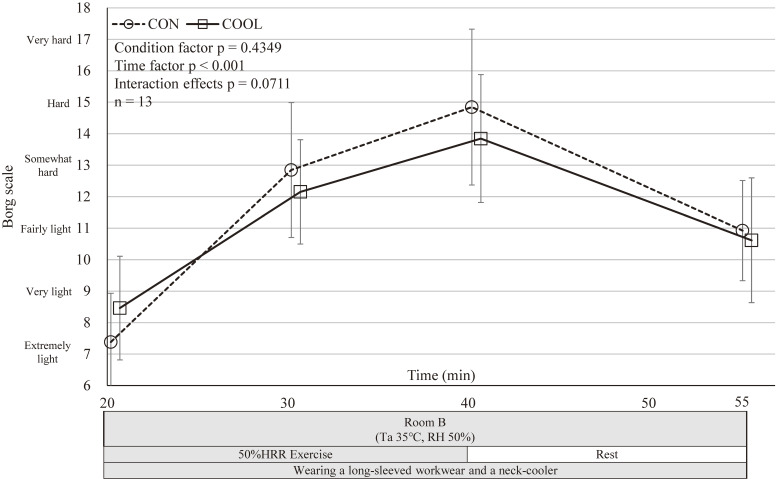
Trend of mean rate of perceived exertion. Mean rate of perceived exertion in CON and COOL conditions; 20 min, immediately before exercise initiation; 30 min, 10 min after exercise initiation; 40 min, 20 min after exercise initiation; 55 min, test completion. Values are mean ± SD (n = 13). CON, control condition; COOL, with neck cooling condition; RH, Relative Humidity; SD, standard deviation; Ta, ambient temperature. No significant differences in a mixed model.

Participants reported significantly cooler sensations in the COOL condition, while RPE did not differ significantly between conditions (Figs. 5–7).

## Discussion

This is the first study to evaluate the effects of neck cooling by measuring core temperature while applying a constant exercise load in a temperature-controlled hot environment. A key strength of this research is the comprehensive evaluation of cooling effects from multiple perspectives, including core temperatures (esophageal and rectal), skin temperature, heart rate, estimated sweat volume, and subjective evaluations, which are often difficult to assess in field settings. By utilizing an artificial climate chamber, we standardized temperature and humidity conditions, ensured consistent clothing and neck cooler usage, and prepared ice under the same conditions. Exercise intensity was set at 50% HRR for each participant, and efforts were made to minimize any variations in participants’ conditions by instructing them on their pre-experimental activities and diet. Furthermore, data deemed inappropriate for analysis based on the post-experiment results were excluded to ensure accuracy.

The cooling effects of neck cooling using ice did not demonstrate significant changes in core temperature, skin temperature, estimated sweat volume, or rating of perceived exertion (RPE) under the COOL condition. Choi et al. [21] reported that applying an ice pack to a limited area on the back of the neck (14 × 11 cm) did not lower rectal temperature (T_re_), but this study noted that the ice pack had melted by 60 minutes into a 120-minute experiment, suggesting insufficient cooling intervention. In contrast, our study covered a larger neck surface area (50 × 7 cm) and maintained cooling throughout the experiment, but no significant effect was observed.

Two potential reasons may explain why no significant cooling effect was observed in this study. The first possibility is that the heat dissipation provided by the neck cooler was insufficient relative to the heat produced during exercise. The second possibility is that a strong negative feedback mechanism regulated by the preoptic area of the hypothalamus, the thermoregulatory center, may have suppressed the body’s heat dissipation mechanisms, such as skin vasodilation and sweating.

Regarding the first reason, the cooling effect of the neck cooler might have been compromised if the evaporation-based heat dissipation was obstructed. The neck cooler used in this study was designed to wrap around the entire neck, potentially hindering sweat evaporation from the neck’s surface and reducing the overall evaporative cooling effect. Another possibility is that the heat dissipation was insufficient due to inadequate contact between the neck cooler and the skin, which may have been necessary to lower the core temperature. Shvartz et al. [23] reported that covering 2.2% of the body surface area with a 3-meter hose to circulate 8.3 °C water around the neck or chest reduced rectal temperature by 0.5 °C and suppressed sweat production by 16–22%. Additionally, Inoue et al. [14] reported that using a chiller vest to circulate 10 °C water effectively reduced esophageal temperature by 0.745 °C and rectal temperature by 0.454 °C when applied to multiple upper body sites, such as the neck, chest, and armpits. Based on these findings, effective neck cooling may require a cooling device that maintains consistent and stable contact with the skin to ensure efficient heat dissipation.

The second reason could be that the negative feedback mechanism, controlled by the preoptic area of the hypothalamus, was excessively activated, thereby inhibiting the body’s heat dissipation responses. The human thermoregulatory center is located in the preoptic area of the anterior hypothalamus [24]. According to Takaki et al. [25], signals from cutaneous thermoreceptors (warm and cold receptors) are transmitted through the spinal cord and lateral parabrachial nucleus to the preoptic area, initiating autonomic thermoregulatory responses such as heat production and dissipation. Temperature perception involves signals being transmitted through the spinothalamic tract to the thalamus and cerebral cortex, leading to conscious behavioral thermoregulation, such as taking off clothes or drinking fluids. While there were no differences in behavior between the CON and COOL conditions in this study, we hypothesize two points that could have affected autonomic thermoregulation.

First, we considered the potential impact of stimulating cold receptors. The head, neck, and face are known to be highly heat-sensitive areas [26], and the number of cold receptors per square centimeter is significantly higher in these regions compared to the torso, with the lips having 15–25 cold receptors per cm^2^ and the torso having 1 or fewer [27]. Therefore, the cooling intervention at the neck may have stimulated these cold receptors, sending a strong cold signal to the preoptic area and activating a negative feedback mechanism that suppressed heat dissipation responses.

Second, we considered the impact of cooling the internal carotid artery. It is known that when the preoptic area, the thermoregulatory center, is warmed, sweating occurs throughout the body [24], and the temperature of the preoptic area itself is also thought to directly influence autonomic thermoregulatory responses. The preoptic area is supplied with blood by the anterior cerebral artery and anterior communicating artery, both of which receive blood from the internal carotid artery. This indicates that arterial blood is directly delivered from the internal carotid artery to the thermoregulatory center, and it has been suggested that cooling the neck could lower brain temperature [23]. Based on this, it is possible that neck cooling reduced the temperature of the preoptic area, strengthening the negative feedback mechanism. Cao et al. [9] conducted a review of studies on neck cooling in the sports field and concluded that, while it is understood that behavioral thermoregulation cannot be employed during exercise, neck cooling has the potential to increase exercise duration and intensity. They explained that this effect occurs by masking the perceived level of heat stress, which may increase the risk of heat-related illness. Similar conclusions have been reported by several other studies [22, 26]. Although it is challenging to directly apply findings from the sports field to the general worker population, these results offer important insights.

Based on these considerations, effective neck cooling requires either maximizing heat dissipation or minimizing excessive stimulation of cold receptors. Increasing cooling intensity could lower core temperature even over a limited surface area, but there is a risk of frostbite, which poses a limitation. Therefore, to suppress the rise in core temperature through neck cooling, it is considered necessary to either maintain close contact with the skin and ensure heat dissipation using sufficiently low temperatures from the neck cooler, or to use a cooling method at an appropriate temperature to prevent excessive negative feedback.

### Limitations

This study has three limitations. First, the participants were limited to healthy Japanese men in their 20s to 30s to ensure a controlled and uniform population for examining physiological and medical effects. Therefore, the findings may not be generalizable to individuals of other age groups, women, or those of different nationalities. Second, the sample size was relatively small, it was determined based on a sample size calculation as described in the Methods section, and the number of participants was finalized with consideration of completing both conditions within a single season without the influence of heat acclimatization. While the statistical power does not reach 90%, it is ensured to be at least 80%. Third, the exercise was performed using a bicycle ergometer focused on lower limb movement, differing from actual labor environments. However, this method was suitable for maintaining consistent workload levels. Lastly, variations in participant activity levels could have influenced the neck cooler’s contact and ice melting rate. However, the neck cooler temperature remained below 10 °C throughout the experiment, indicating consistent cooling.

## Conclusion

This study is the first to investigate the effects of neck cooling as a preventive measure against heat-related illnesses in general workers. Cooling the neck at around 10 °C did not significantly impact core temperature or perceived exertion in workers. When performing neck cooling, it is considered desirable to either maintain close contact with the skin and cool at sufficiently low temperatures to ensure effective heat dissipation, or to cool at an appropriate temperature that prevents excessive negative feedback.

## Abbreviations

BMI: Body Mass Index
CON condition: Control condition (without neck cooling condition)
COOL condition: With neck cooling condition
HR: Heart rate
HRR: Heart Rate Reserve
PCM: Phase Change Material
RH: Relative Humidity
RPE: Rating of perceived exertion
SD: Standard deviation
Ta: Ambient temperature
T_es_: Temperature of esophagus
T_fh_: Temperature of forehead skin
T_re_: Temperature of rectum
VO_2max_: Maximal oxygen consumption

## Supplementary information

CONSORT 2010 Checklist

## Declarations

### Availability of data and material

The datasets used and/or analyzed during the current study are available from the corresponding author on reasonable request.
